# Establishment and validation of cell pools using primary muscle cells derived from satellite cells of pig skeletal muscle

**DOI:** 10.1007/s11626-019-00428-2

**Published:** 2019-12-23

**Authors:** Katharina Metzger, Armin Tuchscherer, Marie-France Palin, Siriluck Ponsuksili, Claudia Kalbe

**Affiliations:** 1 Institute of Muscle Biology and Growth, Leibniz Institute for Farm Animal Biology (FBN), Wilhelm-Stahl-Allee 2, D-18196, Dummerstorf, Germany; 2 Institute of Genetics and Biometry, Leibniz Institute for Farm Animal Biology (FBN), Wilhelm-Stahl-Allee 2, D-18196, Dummerstorf, Germany; 3 Sherbrooke Research & Development Centre, Agriculture and Agri-Food Canada (AAFC), Sherbrooke, Canada; 4 Institute of Genome Biology, Leibniz Institute for Farm Animal Biology (FBN), Wilhelm-Stahl-Allee 2, D-18196, Dummerstorf, Germany

**Keywords:** Cell pool, Satellite cells, Myoblasts, Growth profile, Real-time monitoring

## Abstract

Primary cell cultures derived from satellite cells of skeletal muscle provide an appropriate in vitro model for proliferating myoblasts and differentiating myotubes for muscle biological research. These cell cultures may consist of harvested cells per animal or of a cell pool made of cells from several animals. However, cell pooling reduces the biological variability of the different cell donors. On the other hand, the use of cell pools offers an opportunity to use less donor tissue and to perform long-term projects with a broad spectrum of analysis and replications. In the literature, information about the donors of cell pools, the procedure used for pooling, and the characterization/validation of cell pools is often lacking. In this study, we established three cell pools consisting of M. *rhomboideus* or M. *longissimus* from ten or six piglets, each with one gender and medium birth weight. Real-time impedimetric monitoring was used to evaluate the proliferative growth behavior of myoblasts for the cell pools in comparison to their corresponding unpooled cells over a period of 72 h, with a measurement being taken every 30 min. For each of the tested cell pools, cell index, slope, and doubling time did not differ between the cell pool and the unpooled cells of the donor animals. Differentiation capacity and mRNA expression of *PAX7*, *MYOD* and *MYOG* remained unchanged between the cell pool and the unpooled cells. Current results support that the use of cell pools is an appropriate method to reflect the average proliferative growth behavior of unpooled cells.

The discovery of satellite cells (Mauro 1961) from skeletal muscle, their isolation, and their subsequent cultivation as proliferating and differentiating progenies (myoblasts and myotubes) provides a unique model for muscle biology research. It is known that the adult myogenesis occurring after the activation of satellite cells can be viewed as a suitable model for embryonic myogenesis and that their programmed transcriptional profiles are similar (Parker et al. 2003). Moreover, these *in vitro* systems enable research investigating the direct effects of bioactive compounds (e.g., elderflower extracts (Bhattacharya et al. 2013), phytoestrogens (Kalbe et al. 2008), or fatty acids (McFarland et al. 2011)) on muscle growth and differentiation.

There are two different approaches to isolate satellite cells from skeletal muscle: the direct isolation of satellite cells from digested muscle tissues and the isolation of single muscle fibers. The direct isolation of satellite cells yields more satellite cells. This method was established by Bischoff (1974) using rat muscle and subsequently adapted for farm animals, such as sheep (Dodson et al. 1986), chicken (Yablonka-Reuveni et al. 1987), cattle (Dodson et al. 1987), turkeys (McFarland et al. 1988), fish (Powell et al. 1989), pigs (Doumit and Merkel 1992), and horses (Greene and Raub 1992). The myofiber isolation method is advantageous if it is necessary to maintain the satellite cells in their characteristic position (niche) and in a quiescent state. This method was pioneered by Bekoff and Betz (1977) and Bischoff (1986) using rat skeletal muscle and was later performed with goat and pig muscle fibers (Yamanouchi et al. 2009; Wilschut et al. 2010).

Primary cell cultures of satellite cells derived from skeletal muscle tissue can be performed from one individual animal or as a cell pool consisting of cells from several animals. Cell pooling in itself was and still is a matter of discussion (Stoddart et al. 2012). However, it is generally agreed that this technique offers an opportunity to perform long-term projects with a broad spectrum of experiments, analyses, and multiple replications. In addition, it is known that pooling of cells from multiple donors reduces the biological variability of the different cell donors. Working with cell pools therefore requires a clear and transparent description of their establishment and composition.

In the present study, we used real-time impedimetric cell monitoring to compare the growth profile of three different cell pools of porcine proliferating muscle cells with that of corresponding unpooled cells of several donor pigs. Our objective was to determine whether cell pool growth is similar to the unpooled cells from individual donors.

## Isolation of satellite cells

**Fig. 1:**
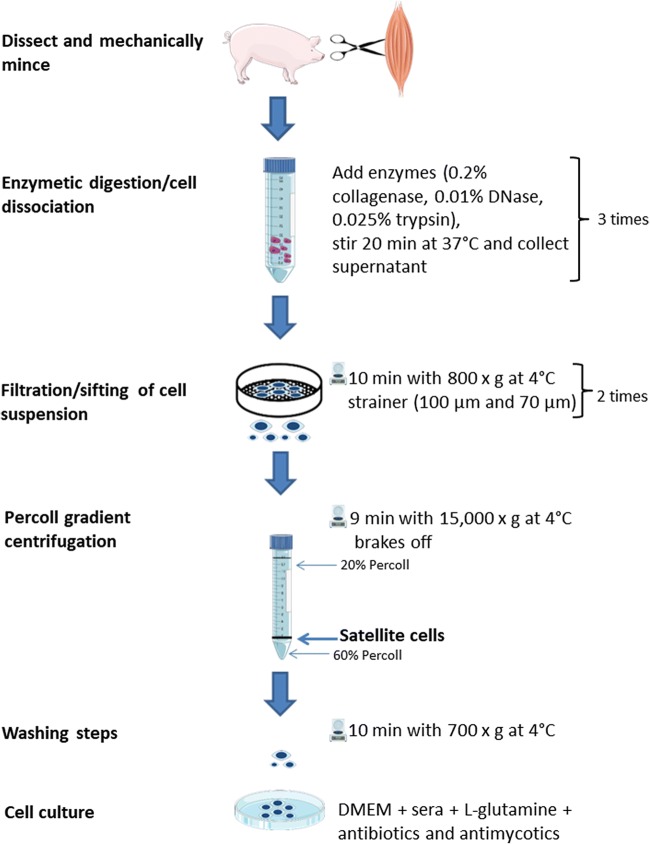
Schematic overview of the satellite cell isolation procedure using porcine skeletal muscle tissue. This procedure is adapted from Mau et al. 2008.

All procedures were in accordance with the German Law of Animal Protection. In this study, we used skeletal muscle tissue from 26 piglets with normal birth weight (1.36 ± 0.15 kg) at three different ages (Pool 1, *M. longissimus*, *n* = 6, Day 4 of age, male; Pool 2, *M. rhomboideus*, *n* = 10, Day 5 of age, female; Pool 3, *M. rhomboideus*, n = 10, Day 20 of age, female). These piglets were from two different research projects (project 1 = Pool 1; project 2 = Pool 2 and 3) that were carried out at the pig-breeding facility of the Leibniz Institute for Farm Animal Biology (FBN, Dummerstorf, Germany). Piglets were killed at the FBN slaughterhouse using exsanguination after captive-bolt pistol (4 and 5 d of age) or electro stunning (20 d of age). The skeletal muscle tissue was dissected and washed in enriched phosphate-buffered saline (PBS-D; 144-mM NaCl, 5.4-mM KCl, 25-mM glucose, 14-mM sucrose, 5-mM Na_2_HPO_4_, 50-IU/mL penicillin, 50-μg/mL streptomycin, and 1-μg/mL phenol red, adjusted to pH 7.4 at 22°C) until the isolation procedure (Fig. 1). The muscle samples (Pool 1: 14.84 ± 0.79 g from *M. longissimus;* Pool 2: 4.24 ± 0.79 g = the whole *M. rhomboideus*, Pool 3: 6.23 ± 1.11 g = the whole *M. rhomboideus*) were trimmed of visible connective tissue, weighed, washed with PBS-D, and minced with scissors. The isolation procedure has been described by Mau et al. 2008, but we have modified the enzymatic digestions and the Percoll gradient centrifugation steps (Fig. 1). Briefly, the cells were dissociated by fractional digestion using a mixture of 0.2% collagenase (Collagenase type I, CLS I, C1–22, Biochrom, Berlin, Germany), 0.01% DNase (DNase I, AppliChem, Darmstadt, Germany), and 0.025% trypsin (Sigma-Aldrich, Taufkirchen, Germany) in Hank’s balanced salt solution (HBSS; Biochrom) for 20 min at 37°C with medium stirring speed. The digestion was then stopped by being placed on ice for 2 min. The supernatant was removed, diluted 1:1 with isolation medium (MEMα (GIBCO Thermo Fisher, Schwerte, Germany) supplemented with 0.2-M L-glutamine (Carl Roth, Karlsruhe; Germany), 100-IU/mL penicillin (Biochrom), 100-μg/mL streptomycin (Biochrom), 2.5-μg/mL amphotericin (Sigma-Aldrich), and 10% fetal bovine serum (FBS; Sigma-Aldrich)), sifted through a 100-μm nylon strainer (Corning, Wiesbaden, Germany) and centrifuged for 10 min at 800 g and 4°C. The supernatant was then discarded, and the pellet was suspended in 5 mL of isolation medium. The remaining digestion solution was replenished with 25 mL of the abovementioned enzyme mixture in HBSS. The procedure was repeated twice. For each animal, the cell suspensions obtained after each digestion were pooled and sifted through a 70-μm nylon strainer. The satellite cells were enriched by Percoll (Sigma-Aldrich, 20% and 60% in PBS) gradient centrifugation. Specifically, 1.5 mL of a 60% Percoll solution was added to a 15-mL Falcon tube, and 11.5 mL of a 20% Percoll solution was then layered on top of the first one. The gradient was finally completed by adding 2 mL of the cell suspension. The gradients were centrifuged at 15,000 g for 9 min at 4 °C with the brakes off. The layer containing the satellite cells (at the interface of the 20% and 60% Percoll solutions, see Fig. 1) was carefully removed, transferred to isolation medium, and centrifuged at 700 g for 10 min at 4 °C. The supernatants were discarded, and the cell pellets were washed twice with isolation medium. Finally, the cell pellet was resuspended, the number of viable cells was determined (Countess, Invitrogen), and approximately 10^5^ cells/cm^2^ were seeded on gelatin-coated (0.1%) 100-mm plastic cell culture dishes (Sarstedt, Sarstedt, Germany) in 15-mL growth medium (DMEM (Biochrom) supplemented with 0.2-M L-glutamine (Carl Roth), 100-IU/mL penicillin (Biochrom), 100-μg/mL streptomycin (Biochrom), 2.5-μg/mL amphotericin (Sigma-Aldrich), 10% FBS (Sigma-Aldrich), and 10% donor horse serum (HS; Sigma-Aldrich)). After 48 h incubation (37 °C, 6% CO_2_), the cells were washed with PBS (Biochrom), and the medium was changed. After 72 h, the cell monolayer was approximately 90 to 95% confluent. These cells were then harvested and cryopreserved as described by Mau et al. 2008.

## Establishment of cell pools

For the establishment of cell pools, approximately 3 × 10^6^ satellite cell progenies of each animal were pooled. For Pool 1, the cells were grown for 24 h (approximately 90% confluent). Pools 2 and 3 were established with the aim of obtaining the largest possible cell pools. Therefore, the cells were grown until reaching 90% confluence and then split in a ratio of 1:3 and harvested upon reaching a confluence of 90%. Cells were aliquoted (2 × 10^6^ cells per vial) and cryopreserved at passage number two for Pool 1 and three for Pools 2 and 3.

**Fig. 2:**
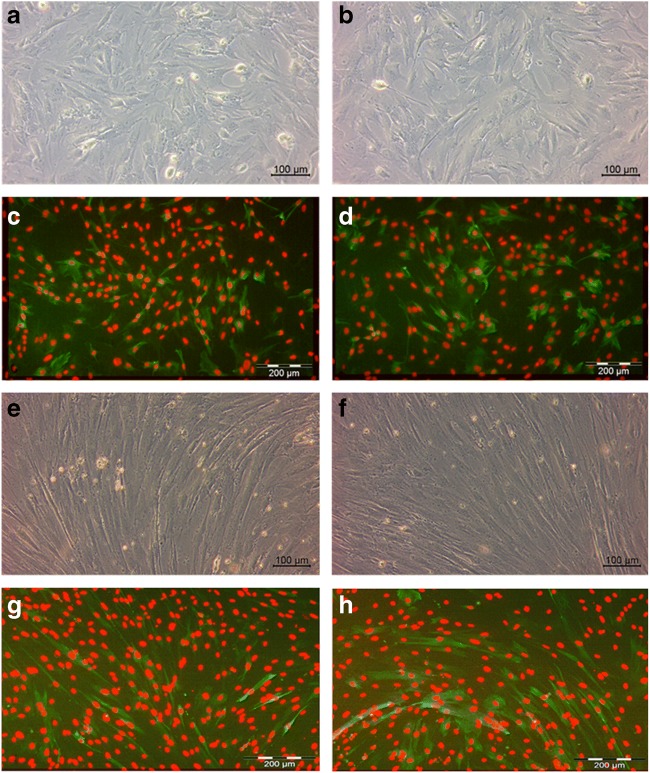
Cell pools derived from satellite cells of *M. rhomboideus* at 5 (*a, c, e, g*) or 20 d (*b, d, f, h*) of age. Myoblasts were seeded on gelatin-coated dishes and grow for 24 h (*a, b*). Desmin-positive cells (*c, d*, in *green*) were determined by immunostaining using a D1033 mouse monoclonal anti-desmin antibody. The stained nuclei appeared red (DAPI). Pool 2 (*c*) and Pool 3 (*d*) exhibited 98 ± 1% and 95 ± 2% desmin positive cells, respectively (> 8000 cells per pool were analyzed). Myoblasts were seeded on Geltrex™ (growth factor reduced, 1:100)-coated dishes and allowed to grow and differentiate for 11 d (*e, f*). Pool 2 (*g*) and Pool 3 (*h*) exhibited 53 ± 1% and 55 ± 1% differentiating myotubes. A myotube was defined as a desmin-positive cell containing three or more nuclei (DAPI). Ten representative pictures from each cell pool or corresponding unpooled cells were analyzed (Q-win imaging system, Leica).

For each cell pool, a cell aliquot (1 × 10^6^ cells) was taken and seeded on a gelatin-coated 100-mm cell culture dish (Sarstedt) for the estimation of the percentage of myogenic cells by immunostaining using an antibody against desmin (D1033 mouse monoclonal anti-desmin antibody, Sigma-Aldrich), which is characteristic of replicating myoblasts (Kaufman and Foster 1988) and 4,6-diami-dino-2-phenylindole (DAPI, Carl Roth) for the nuclei. After approximately 24 h, the cells were harvested upon reaching a confluence of 80% (Fig. 2*a*, *b*). Cell fixation and immunostaining were performed according to Mau et al. (2008) and analyzed using the Q-Win imaging software (Leica, Wetzlar, Germany) on at least 8000 cells per pool. In the two representative pictures (Fig. 2*c*, *d*), desmin positive cells are in green and the nuclei in red (Pool 1, 97 ± 1%; Pool 2, 98 ± 1%; Pool 3, 95 ± 2%).

## Myogenic phenotype of pooled vs. unpooled cells

The myogenic phenotype was determined by the mRNA expression of the satellite cell marker *PAX*7 (paired box transcription factor 7) and the muscle-specific transcription factors *MYOD* (myogenic differentiation factor) and *MYOG* (myogenin) after 72 h of proliferation. Therefore, for each cell pool and their corresponding unpooled cells, a cell aliquot (1 × 10^6^ cells) was taken and seeded on a gelatin-coated 100-mm cell culture dish (Sarstedt) with growth medium for 72 h. The RNA isolation (Kalbe et al. 2008), the reverse transcription, and real-time PCR procedures, including primer information (Kalbe et al. 2018), were previously described. Data are expressed as arbitrary units after normalization with the endogenous reference gene *HPRT1* (hypoxanthine phosphoribosyltransferase 1). There were no differences between the cell pools and their corresponding unpooled cells with regard to the mRNA expression of *PAX7* (Pool 1, 0.034 ± 0.011 vs. 0.025 ± 0.004, *P* = 0.48; Pool 2, 0.005 ± 0.004 vs. 0.003 ± 0.001; *P* = 0.59; Pool 3, 0.003 ± 0.002 vs. 0.004 ± 0.001, *P* = 0.86). Moreover, the mRNA expression of *MYOD* (Pool 1, 0.100 ± 0.072 vs. 0.130 ± 0.030, *P* = 0.72; Pool 2, 0.011 ± 0.007 vs. 0.005 ± 0.002; *P* = 0.41; Pool 3, 0.004 ± 0.005 vs. 0.005 ± 0.002, *P* = 0.93) and *MYOG* (Pool 1, 0.989 ± 0.333 vs. 0.881 ± 0.136, *P* = 0.78; Pool 2, 0.053 ± 0.020 vs. 0.016 ± 0.007; *P* = 0.13; Pool 3, 0.024 ± 0.012 vs. 0.010 ± 0.004, *P* = 0.30) did not differ between cell pool and their corresponding unpooled cells.

To estimate the differentiation capacity, 4 × 10^5^ cells per cell pool or unpooled cells were seeded in Geltrex™ (growth factor reduced, 1:100, Gibco Thermo Fisher)-coated 100-mm cell culture dishes. Cells were grown in growth medium for 4 d, in growth medium 2 (DMEM (Biochrom) supplemented with 0.2-M L-glutamine (Carl Roth), 100-IU/mL penicillin (Biochrom), 100-μg/mL streptomycin (Biochrom), 2.5-μg/mL amphotericin (Sigma-Aldrich), 10% FBS (Sigma-Aldrich), and 1-μM insulin (Sigma-Aldrich)) for 1 d and then in serum-free differentiation medium (MEM (Biochrom) supplemented with 0.2-M L-glutamine (Carl Roth), 100-IU/mL penicillin (Biochrom), 100-μg/mL streptomycin (Biochrom), 2.5-μg/mL amphotericin (Sigma-Aldrich), 1-μM insulin (Sigma-Aldrich), 1-μM cytosine β-D-arabinofuranoside (Sigma-Aldrich), 0.5-mg/mL bovine serum albumin (Sigma-Aldrich), 0.1-nM dexamethasone (Sigma-Aldrich), 0.5-μg/mL linoleic acid (Sigma-Aldrich), and 100-μg/mL transferrin (bovine holoform, Sigma-Aldrich)) for 6 d. The estimation of fusion degree was performed after 6 d of differentiation as described by Mau et al. 2008 (Fig. 2*e–*h). A myotube was defined as three or more nuclei in a cell membrane. There were no significant differences in the fusion degree between each cell pool (1, 2, and 3) and their corresponding unpooled cells (Pool 1, 32.89 ± 1.69% vs. 29.16 ± 4.13%, *P* = 0.44; Pool 2, 52.74 ± 1.20% vs. 50.04 ± 3.59%, *P* = 0.50; Pool 3, 54.91 ± 1.14% vs. 51.34 ± 3.60%, *P* = 0.37). The observed fusion degrees are in agreement with other porcine studies (Doumit and Merkel 1992; Baquero-Perez et al. 2012).

## Comparison of pooled vs. unpooled cells

**Fig. 3:**
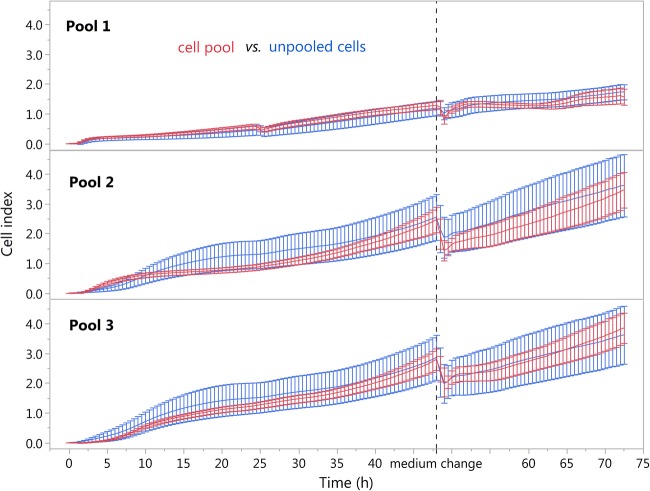
Cell index (means ± standard deviation) for three different cell pools (Pool 1–3) and their corresponding unpooled cells was measured in real time every 30 min over 72 h using the xCELLigence system (ACEA biosciences Inc). The cell index is a dimensionless value that measures the relative change in electrical impedance to represent the cell status. The cell pools are shown in *red* and represent the whole unpooled cells from the corresponding animals shown in *blue*.

Real-time myoblast proliferation was monitored by recording the impedance every 30 min. This monitoring was carried out with the xCELLigence RTCA SP system, using 96-well culture plates with electrodes in the bottom of each well (e-plate 96, ACEA Biosciences Inc., San Diego, CA). Data are presented as the cell index (arbitrary units), which corresponds to the changes in impedance over a specific time period, in our case, a 72-h growing period. Impedance as a cellular readout was previously established (Giaever and Keese 1993) and previously used for primary skeletal muscle myoblasts (Sente et al. 2016). Most publications using impedance-based label-free technology have focused on the effects of various compounds on cellular adhesion and proliferation (Atienzar et al. 2011; Will et al. 2012). For all experiments, the cells were thawed rapidly in a water bath at 37 °C and washed with growth medium. The e-plates were coated with gelatin, and 5000 (Pool 1 – *M. longissimus*) or 4000 (Pool 2 and 3 – *M. rhomboideus*) cells were seeded per well using growth medium. For experiments with unpooled cells from individual animals of Pool 1, the cells were seeded in two wells (duplicate), and cell pool 1 was present in four wells. The experiment was repeated 6 times with varying plate positions. After 24 h, half of the culture medium was changed, and a complete medium change was performed after 48 h. For Pool 2 and Pool 3, three different xCELLigence runs were performed. For each run, three wells were seeded with unpooled cells from each animal and 10 wells per pool. The medium was changed after 48 h. During all xCELLigence runs, 14 individual wells were excluded because of erroneous values. The cell indices profile (given as mean ± standard deviation) over the 72-h growth period is shown in Fig. 3. For each of the three cell pools, the cell index profiles of pooled cells were similar to those of the unpooled cells from the corresponding pig donors. Therefore, these results suggest that the use of pooled cells is an appropriate method to reflect the average proliferative growth behavior of unpooled cells. For statistical analysis, data were subjected to analysis of variance using the mixed procedure in SAS (Version 9.2, SAS Inst Inc., Cary, NC). Samples (cell pool or unpooled cells) and the replication of the experiment (six for Pool 1 and three for Pool 2 and 3) were used as fixed factors. Differences between the least squares means were tested with Tukey tests. There were no significant differences between the average cell index values of each cell pool (1, 2, and 3) and their corresponding unpooled cells (Pool 1, 0.855 ± 0.150 vs. 0.818 ± 0.061, *P* = 0.83; Pool 2, 1.484 ± 0.386 vs. 1.706 ± 0.129, *P* = 0.60; Pool 3, 1.766 ± 0.357 vs. 1.929 ± 0.119, *P* = 0.68). In agreement with these findings, the cell indices of the cell pools and their corresponding unpooled cells were similar in each experimental replication (Pool 1, *P* = 1.00; Pool 2, *P* ≥ 0.76, Pool 3, *P* ≥ 0.98, data not shown). The slope (1/h) describes the steepness and incline of the cell index curve and is an indication of the growth rate. In this study, the slope was calculated over the experimental period of 72 h. The averaged slope values were unchanged between the unpooled cells of the animals and the corresponding cell pools (Table 1, Pool 1, *P* = 0.34, Pool 2, *P* = 0.73; and Pool 3, *P* = 0.46). Moreover, for each experimental replication, there was no difference in slope values between the unpooled cells of the animals and their corresponding pools (Table 1, *P* ≥ 0.94). The doubling time (h) is also an indicator for proliferative potential of the cells describing the period of time required for the cell index to double. Doubling time was calculated over a 67 h period (from 5 h to 72 h), starting at 5 h to allow the myoblasts to attach after seeding. The average doubling times were also unchanged between the unpooled cells and their corresponding cell pools (Table 2, Pool 1, *P* = 0.09; Pool 2, *P* = 0.88; and Pool 3, *P* = 0.36). In addition, for each experimental replication, there was no difference in doubling time between the unpooled cells and their corresponding pools (Table 2, *P* ≥ 0.34). The current study results clearly show that it would be appropriate to use the three different cell pools in different experimental setups because they perfectly reflected their corresponding six (Pool 1) or ten (Pool 2 and 3) donor piglets based on the real-time monitoring of growth behavior. To ensure that all donors respond to the same extent within their individual variability, we therefore recommend carefully establishing representative muscle cell pools derived from satellite cells of muscle tissue from several donors. In conclusion, the following requirements are indispensable when using cell pools derived from several donors: (1) a detailed data record for the donor’s background including the number of animals and their gender, donor tissue (specific muscle), and birth weight, which is known to strongly affect myogenesis (Paredes et al. 2013); (2) a detailed description of the isolation and establishment procedures for the cell pools. It is also important to mention that the seeded cell number and the developmental stage (cell passage number) for each donor animal should always be equal; (3) a minimum of three experimental replicates is needed to minimize variations.

**Table 1:** Comparison of slopes over a growing period of 72 h for pooled porcine myoblasts and their corresponding unpooled cells

**Pool**	**Donor tissue**	**Experimental Replication**	**Slope (1/h)**^**a**^**:**		***P***
			**Cell pool**	**Unpooled cells**	
Pool 1	*M. long.*, 4 d	1	0.024 ± 0.003	0.026 ± 0.001	1.00
		2	0.024 ± 0.003	0.025 ± 0.001	1.00
		3	0.019 ± 0.004	0.025 ± 0.002	0.94
		4	0.022 ± 0.003	0.025 ± 0.001	0.99
		5	0.021 ± 0.003	0.023 ± 0.001	1.00
		6	0.020 ± 0.004	0.025 ± 0.002	0.99
		average	0.022 ± 0.003	0.025 ± 0.001	0.34
Pool 2	*M. rhom.,* 5 d	1	0.038 ± 0.012	0.034 ± 0.004	1.00
		2	0.034 ± 0.010	0.042 ± 0.004	0.97
		3	0.051 ± 0.009	0.056 ± 0.003	1.00
		average	0.041 ± 0.008	0.044 ± 0.003	0.73
Pool 3	*M. rhom.,* 20 d	1	0.044 ± 0.007	0.038 ± 0.003	0.94
		2	0.045 ± 0.007	0.041 ± 0.003	0.99
		3	0.056 ± 0.005	0.054 ± 0.002	1.00
		average	0.048 ± 0.005	0.044 ± 0.002	0.46

^a^The slope was calculated with the xCELLigence (ACEA Biosciences Inc) software (RTCA, Version 1.2.1) using the following formula: cell index = slope * time + intercept and is presented as least squares means ± standard errors

*M. long.*, *M. longissimus*; *M. rhom., M. rhomboideus*; *P*, *P* value of Tukey test

**Table 2:** Comparison of doubling time over a growing period from 5 to 72 h for pooled porcine myoblasts and their corresponding unpooled cells

**Pool**	**Donor tissue**	**Experimental Replication**	**Doubling time (h)**^**a**^**:**		***P***
			**Cell pool**	**Unpooled cells**	
Pool 1	*M. long.*, 4 d	1	24.00 ± 2.82	19.36 ± 1.20	0.92
		2	22.68 ± 3.18	20.48 ± 1.36	1.00
		3	23.22 ± 2.27	19.29 ± 0.97	0.90
		4	24.78 ± 3.31	20.39 ± 1.41	0.98
		5	25.75 ± 2.33	19.23 ± 0.99	0.34
		6	24.66 ± 2.96	18.07 ± 1.26	0.66
		average	24.18 ± 2.02	19.47 ± 0.86	0.09
Pool 2	*M. rhom.,* 5 d	1	25.19 ± 4.62	22.70 ± 1.64	1.00
		2	25.26 ± 4.53	25.82 ± 1.54	1.00
		3	22.89 ± 3.53	23.24 ± 1.12	1.00
		average	24.45 ± 3.35	23.92 ± 1.15	0.88
Pool 3	*M. rhom.,* 20 d	1	19.77 ± 2.64	24.30 ± 1.53	0.68
		2	24.86 ± 4.44	28.70 ± 2.22	0.97
		3	25.48 ± 3.86	25.52 ± 1.93	1.00
		average	23.37 ± 2.55	26.18 ± 1.30	0.36

^**a**^The doubling time was calculated with the xCELLigence (ACEA Biosciences Inc) software (RTCA, Version 1.2.1) using the following formula: cell index = A * 2^(t/CI doubling-time) and is presented as least squares means ± standard errors

*M. long.*, *M. longissimus*; *M. rhom., M. rhomboideus*; *P,* P value of Tukey test
